# Cargo and Dynamin Regulate Clathrin-Coated Pit Maturation

**DOI:** 10.1371/journal.pbio.1000057

**Published:** 2009-03-17

**Authors:** Dinah Loerke, Marcel Mettlen, Defne Yarar, Khuloud Jaqaman, Henry Jaqaman, Gaudenz Danuser, Sandra L Schmid

**Affiliations:** Department of Cell Biology, The Scripps Research Institute, La Jolla, California, United States of America; Princeton University, United States of America

## Abstract

Total internal reflection fluorescence microscopy (TIR-FM) has become a powerful tool for studying clathrin-mediated endocytosis. However, due to difficulties in tracking and quantifying their heterogeneous dynamic behavior, detailed analyses have been restricted to a limited number of selected clathrin-coated pits (CCPs). To identify intermediates in the formation of clathrin-coated vesicles and factors that regulate progression through these stages, we used particle-tracking software and statistical methods to establish an unbiased and complete inventory of all visible CCP trajectories. We identified three dynamically distinct CCP subpopulations: two short-lived subpopulations corresponding to aborted intermediates, and one longer-lived productive subpopulation. In a manner dependent on AP2 adaptor complexes, increasing cargo concentration significantly enhances the maturation efficiency of productive CCPs, but has only minor effects on their lifetimes. In contrast, small interfering RNA (siRNA) depletion of dynamin-2 GTPase and reintroduction of wild-type or mutant dynamin-1 revealed dynamin's role in controlling the turnover of abortive intermediates and the rate of CCP maturation. From these data, we infer the existence of an endocytic restriction or checkpoint, responsive to cargo and regulated by dynamin.

## Author Summary

Clathrin-mediated endocytosis is the major pathway for the uptake of molecules into eukaryotic cells and is regulated by the GTPase dynamin. Adaptor proteins recruit clathrin to the plasma membrane, where clathrin-coated pits capture transmembrane cargo molecules, again via adaptors. The pits invaginate and pinch off to form clathrin-coated vesicles that carry the cargo into the cell. Live cell imaging has revealed striking heterogeneity in the dynamic behavior of clathrin-coated pits associated with the plasma membrane, yet the nature of this heterogeneity and its functional implications are unknown. We used particle-tracking software to establish an unbiased and complete inventory of the trajectories of clathrin-coated pits visible by total internal reflection fluorescence microscopy. Through statistical analyses, we identified three dynamically distinct subpopulations of coated pits: two short-lived subpopulations corresponding to aborted intermediates, and one longer-lived productive subpopulation. The proportion of each subpopulation and their lifetimes respond independently to molecular perturbations. As a result of systematic modulation of cargo concentration, adaptor levels, and analysis of dynamin mutants, we postulate the existence of an endocytic restriction or checkpoint that governs the rate of clathrin-mediated endocytosis by gating the maturation of clathrin-coated pits.

## Abstract 3

Analysis of live-cell images reveals that clathrin-mediated endocytosis proceeds via distinct subpopulations of clathrin-coated pits, and suggests that an endocytosis check-point is regulated by dynamin and responsive to cargo load.

## Introduction

Clathrin-mediated endocytosis (CME) is the major endocytic pathway in eukaryotic cells. It occurs via clathrin-coated pits (CCPs) that are assembled from cytosolic coat proteins. CCPs capture transmembrane cargo molecules, invaginate, and then pinch off to form clathrin-coated vesicles (CCVs). CME is a constitutive, yet highly regulated process. Biochemical assays of endocytosis score ligand uptake and measure only the ensemble average of successful internalization events, thereby obscuring critical, rate-limiting early stages of maturation and alternative outcomes that might cause variability in individual CCP dynamics. Indeed, live cell imaging has revealed striking heterogeneity in the dynamic behavior of plasma membrane–associated CCPs [1–5].

An important parameter for analyzing CCP heterogeneity is their lifetimes. The lifetime of an individual CCP at the plasma membrane, i.e., the time required for (1) coat initiation, (2) coat propagation, (3) neck constriction, and (4) vesicle budding, is critical for understanding CME. Changes in lifetimes caused by specific molecular perturbations can reveal mechanisms that regulate each of these steps. However, selective probing of all stages of CCP maturation is only possible by mild perturbation of the underlying molecular processes. Detection and interpretation of these necessarily milder phenotypes requires sensitive and comprehensive analysis of individual CCP lifetimes and behavior. To this end, we have employed total internal reflection fluorescence microscopy (TIR-FM), the premier assay to detect early intermediates in CCV formation and visualize the dynamics of CCPs in living cells [1,3–9]. By selectively exciting fluorophores associated with molecular components of CCPs at the ventral plasma membrane, TIR-FM provides excellent signal-to-background ratio and high time resolution. In spite of these strengths, it has remained a challenge to extract reliable measurements of CCP lifetimes from TIR-FM videos. Lifetime measurements are notoriously susceptible to tracking errors, which typically break CCP trajectories into two or more subtrajectories, leading to systematic bias of lifetimes towards shorter values. As a result, tracking has previously been accomplished either manually for a low number of well-discernable, high-intensity CCPs [1,6], or using semiautomated tracking restricted to isolated CCPs, for which no close neighbors are likely to confuse the tracking algorithm [2,4]. Both approaches sample the behavior of arbitrary and typically small subpopulations with relatively uniform properties. To solve these problems and to better exploit the heterogeneity of CCP dynamics as a source of mechanistic information, we have employed particle-tracking software [10] capable of detecting and tracking all CCPs visualized by TIR-FM in an unbiased fashion. Automated detection and tracking enabled analysis of several tens of thousands of trajectories per condition, 100 times more than previous studies, thus providing a comprehensive and accurate measurement of CCP lifetime distributions.

## Results

### Three Kinetically Distinct Subpopulations of CCPs

**Figure 1:**
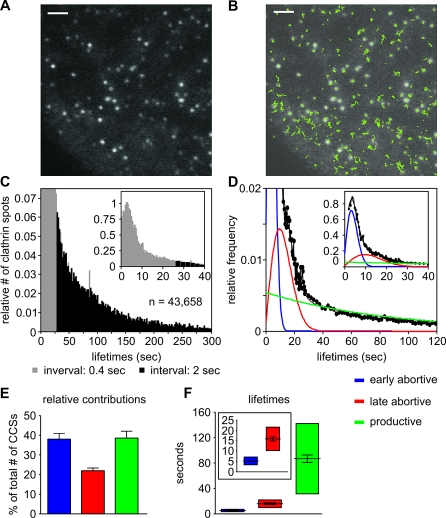
Automated Tracking and Statistical Analyses Detect Three Subpopulations of CCPs at the Plasma Membrane (A) A single frame from a 10-min video of BSC1 cells stably expressing EGFP-labeled clathrin LCa-EGFP (the scale bar indicates 2 μm). (B) Overlay of all trajectories observed during the video. (C) Lifetime distribution for CCPs determined in fast- (grey) and slow-acquisition (black) TIR-FM time-lapse videos. The distribution was determined from the elapsed time between the appearance and disappearance of fluorescent structures present in the time series. *n* = 43,568 CCP trajectories from 23 cells. (D) Identification of three subpopulations of CCPs. Black dots and smoothed black line show distribution of all CCP lifetimes detected by TIR-FM. These data were best fit by three kinetically distinct subpopulations termed early abortive (blue line), late abortive (red line), and productive CCPs (green line). (E and F) Relative contributions and lifetimes of subpopulations. Error bars represent cell-to-cell variation; the height of the lifetime bar in (F) denotes the t50-spread of the distribution, i.e., the range around the characteristic lifetime that contains 50% of the data. Insets in (C, D, and F) show the data at different scales to emphasize short-lived CCP populations.

We used TIR-FM and our automated tracking assay [10] (see Materials and Methods, Figure S1, and Videos S1, S2, and S4) to obtain large and unbiased datasets of CCP dynamics in well-characterized BSC1 cells expressing a fully functional enhanced green fluorescent protein (EGFP)-tagged clathrin light chain a (LCa-EGFP; Figure 1A and 1B) [2]. To capture both fast events at the timescale of seconds and slower events at the timescale of minutes, we combined data from time-lapse sequences taken at a frame rate of 0.4 s for ≥3 min with data from sequences taken at a frame rate of 2 s for 10–15 min (see Materials and Methods). Objects not detected for at least five consecutive frames were not counted, to exclude transient, highly motile structures. Nonetheless, CCPs displayed a nearly exponential decay of lifetimes (Figure 1C), indicating that a large number appear and disappear on the timescale of a few seconds. To further analyze CCP lifetimes, the raw data were fitted to a series of models that differed in the number and types of subdistributions. Our goal was to identify the minimal number of kinetically distinct subpopulations that could account for the overall lifetime distribution observed (see Figure S2A). Model selection was achieved by three strategies. The first two involved minimization of the Bayesian Information Criterion (BIC) [11,12], which defines the optimal tradeoff between the goodness of fit of the model and the number of free model parameters. The application of the BIC requires a priori knowledge of the distribution of experimental errors, which is unknown for lifetime data. Thus, we performed BIC minimization, first, in a fit of the cumulative lifetime histogram, i.e., with the lifetime and the cumulative frequency as the independent and dependent, error-perturbed variables, respectively; and second, in a fit of the inverse of the cumulative histogram, i.e., with the percentage rank as the independent and the lifetime as the dependent error-perturbed variables. In both cases, we approximated the distribution of the fitting errors as normal. The third strategy for model selection involved a nonparametric test of the distribution of the fitting residuals, which did not require a priori assumptions (see Figure S2).

All three strategies identified three statistically significant subpopulations (Figure 1D) with distinct time constants, but broad and overlapping lifetime distributions (Figure 1F). Importantly, at the noise level of our TIR-FM images, accurate assignment of these subpopulations required analysis of >5,000 trajectories (see Figure S2C), and hence, our results strongly relied on the accurate and automatic tracking of all CCPs in multiple videos per experimental condition (see Materials and Methods). Our data contained two short-lived subpopulations with time constants of 5.2 ± 0.1 s and 15.9 ± 1 s, respectively (±jackknifed cell-to-cell error, see Table S1) that were best fit with Rayleigh distributions, i.e., the shortest and longest lifetimes within the population occur less frequently than the intermediate ones. This suggests that our time sampling of 0.4 s per frame was sufficient to capture all events of significant clathrin coat accumulation. A single longer-lived subpopulation (time constant 86.9 ± 5.8 s) was best approximated by an exponentially decaying distribution. Given its long time constant, accurate measurements of the mean lifetime of this population required imaging for ≥10 min. The longer-lived subpopulation was designated the “productive population,” because (1) its kinetics match those of surface-bound transferrin internalization measured biochemically (*t*_1/2_ ≈ 104 s; Figure S3A), and (2) manual tracking of 450 CCPs (for which internalization was confirmed by sequential disappearance from the TIR-FM and the epifluorescence microscopy (EPI-FM) field [5,6]) also yielded a mean lifetime of approximately 100 s (Figure S3B). Accordingly, we hypothesized that the two shorter-lived species correspond to transient, nonproductive events and therefore termed them “early abortive” and “late abortive” CCPs, respectively.

In BSC1 cells, the productive population constituted only 38.6 ± 3.4% of total CCPs at the plasma membrane, with early and late abortive CCPs representing 38.1 ± 3.1% and 21.9 ± 1.4%, respectively (±jackknifed cell-to-cell error; Figure 1E, Table S2). Taking into account the different mean lifetimes and relative contributions of the three individual subpopulations, the mean lifetime of all CCPs is 39 s, much shorter than the half-time of transferrin (Tfn) uptake. This further supports the hypothesis that the two short-lived subpopulations are abortive and do not contribute to Tfn uptake.

To determine whether the existence of these three subpopulations was affected by the nature of the fluorescent tag or the cell type used, we performed lifetime analyses on BSC1 and HeLa cells transiently expressing LCa-tomato and NIH3T3 cells stably expressing LCa-DsRed. We consistently observed one long-lived and two short-lived populations (Table S1B), suggesting that this categorization into kinetically distinct CCP populations is a universal phenomenon of CME. As described by others [4,13], however, we also observed in both HeLa cells and NIH3T3 cells, a higher proportion of larger clathrin-coated structures (CCSs) from which multiple CCSs emerge and disappear. These so-called “nonterminal” events are rarely detected in BSC1 cells, consistent with previous findings [2].

**Figure 2:**
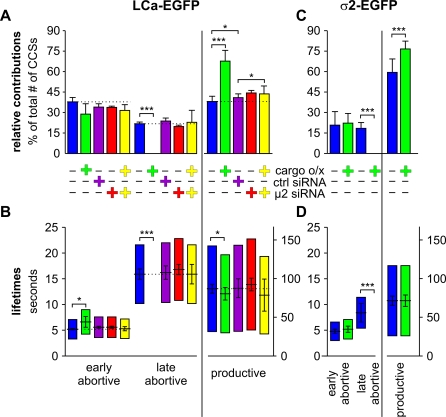
Cargo Concentration Regulates the Efficiency of CCP Maturation, but Is Not Rate-Limiting for CCV Formation Relative contributions and lifetimes of the three CCP populations reported by LCa-EGFP (A and B) or the AP2 rat brain σ2-subunit (σ2)-EGFP (C and D), in control cells, after overexpression of TfnR (cargo o/x) and/or treatment with μ2 siRNA to reduce AP2 levels; a control experiment with nontargeting siRNA is also included. Cargo overexpression increases the relative contribution of productive CCPs at the expense of abortive structures, in an AP2-dependent manner. A single asterisk (*) and triple asterisks (***) indicate confidence levels of *p* < 0.05 and *p* < 10^−4^, respectively (KS-test). The number of CCP trajectories (*n*) and cells (*k*) for each condition are: LCa-EGFP: − cargo o/x (*n* = 43,658, *k* = 23); + cargo o/x (*n* = 23,626, *k* = 18); ctrl siRNA (*n* = 9,376, *k* = 10); σ2 siRNA (*n* = 29,106, *k* = 27); σ2 siRNA + cargo o/x (*n* = 11,071, *k* = 12). σ2-EGFP: − cargo o/x (*n* = 22,252, *k* = 21); + cargo o/x (*n* = 14,100, *k* = 16).

We also analyzed CCP lifetimes by tracking the adaptor protein, AP2, in BSC1 cells expressing the EGFP-tagged σ2 subunit (σ2-EGFP), shown not to interfere with AP2 function [2] (Videos S3 and S5). Our model selection again identified three kinetically distinct populations of σ2-containing CCPs (Figure 2C and 2D, Tables S1 and S2); the preference for three versus two subpopulations was weaker but still highly significant (*p* < 10^−4^ as compared to *p* < 10^−10^ in LCa-EGFP–expressing cells). This further supports our conclusion that each of these subpopulations represents bona fide plasma membrane–associated CCPs rather than clathrin-bearing endosomal structures transiently approaching the cell surface. The percentage of productive CCPs with σ2-EGFP labeling was higher than those labeled with LCa-EGFP (56.3 ± 10.1% as compared to 38.6 ± 3.4%; compare Figure 2A and 2C, Table S2), confirming previous suggestions [2] that adaptors enhance the maturation efficiency of CCPs. The characteristic lifetimes of early abortive CCPs labeled with σ2-EGFP (4.8 ± 0.4 s) were similar to those observed for LCa-EGFP (5.2 ± 0.1 s; compare Figure 2B and 2D, Table S2), suggesting that this very short-lived subpopulation represents stochastic coated pit nucleation events, perhaps triggered by low-affinity interactions between AP2 and phosphatidylinositol-4,5-bisphosphate at the plasma membrane. In contrast, the characteristic lifetimes of both late abortive and productive subpopulations of σ2-EGFP–labeled CCPs (8.4 ± 1.8 s and 71.5 ± 6 s, respectively) were significantly shorter than their LCa-EGFP–labeled counterparts (15.9 ± 1 s and 86.9 ± 5.8 s, respectively). This observation is consistent with findings of others, reporting a general shift of AP2-containing structures toward shorter lifetimes [2,8], although interpretations have varied. It may reflect dissociation of AP2 complexes from CCPs prior to clathrin [8] and/or a nonuniform distribution of AP2 complexes in the clathrin lattice resulting in their differential illumination by the TIRF field [9]. The former interpretation would be consistent with recent findings that Sar1p and the Sec23/24 adaptors can dissociate from budding vesicles prior to the Sec13/31p-containing outer shell of the COPII coat [14,15] and the notion that multivalent clathrin interactions dominate over AP2 interactions at later stages of CCV formation [16].

### Cargo Concentration Enhances CCP Maturation Efficiency

**Figure 3:**
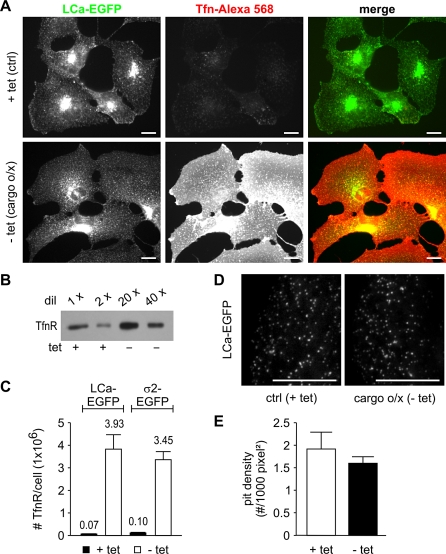
Cargo Overexpression in a Tetracycline (tet)-Repressed System BSC1 cells, coinfected with adenoviruses coding for a tet-repressible transcription activator and the human transferrin receptor (TfnR), were cultured in the presence (+) or absence (−) of tet. (A) Validation of infection efficiency by adenoviruses: cells were loaded with Tfn-Alexa 568, and analyzed by epifluorescence. The scale bars represent 10 μm. (B) Whole-cell lysates were analyzed by SDS-PAGE and western blot. (C) Quantitative analysis of surface-bound TfnRs in BSC1 cells stably expressing LCa-EGFP and σ2-EGFP under control (+ tet) or cargo overexpressing (− tet) conditions. Error bars represent standard deviations of three independent experiments. (D) CCPs, visualized by LCa-EGFP and TIR-FM, in control (ctrl) and TfnR overexpressing (cargo o/x) cells. The scale bar represents 5 μm. (E) Average pit density in LCa-EGFP– and σ2-EGFP–expressing cells under control and cargo overexpressing conditions.

Using semiautomated analysis, Ehrlich et al. [2] previously detected a single, short-lived subpopulation of CCPs in BSC1 cells, which displayed a mean lifetime and relative contribution similar to our late abortive population. In addition, they reported that cargo-associated CCPs rarely failed to proceed to completion, leading to the suggestion that cargo might stabilize abortive CCPs. However, a direct link between CCP maturation and cargo load has not been established. To test the hypothesis that CCP maturation is responsive to the concentration of cargo, we infected BSC1 cells with an adenovirus coding for the human transferrin receptor (TfnR) in a tetracycline (tet)-repressible system. The TfnR is constitutively internalized even in the absence of ligand [17] and thus serves as a model transmembrane cargo molecule. Removal of tet induced TfnR overexpression by >30-fold in nearly all cells (Figure 3A–3C). There have been conflicting observations regarding the effect of TfnR overexpression on surface density of CCPs [18–20]. We confirmed previous biochemical measurements [20] showing that at high levels of TfnR overexpression, the endocytic machinery becomes saturated, i.e., the bulk endocytic efficiency of TfnR declines (Figure S3A), thus obscuring any effect that cargo might have on CCP dynamics. In addition, we did not observe an increase in membrane recruitment of either clathrin or the TfnR adaptor protein AP2, as measured by subcellular fractionation and western blot analysis (Figure S3C and S3D) or by TIR-FM (Figure 3D and 3E).

In cells overexpressing TfnRs, we could no longer detect a significant (*p* > 0.05) late abortive subpopulation labeled with LCa-EGFP, and this population was completely undetectable in σ2-EGFP labeled cells. The contribution of the productive population increased to 67.9 ± 7.9% of LCa-EGFP–labeled CCPs (Figure 2A) and 76.8 ± 5.6% of σ2-EGFP–containing CCPs (Figure 2C). From this, we conclude that CCPs mature with their highest efficiency when a threshold amount of AP2 and cargo are incorporated into the nascent clathrin lattice. In contrast, the relative contribution of early abortive CCPs is unchanged by cargo overexpression further supporting the notion that these are transient structures assembled in a cargo-independent manner.

The mean lifetime of the productive population was only slightly decreased upon cargo overexpression when compared to control conditions (Kolmogorov-Smirnov test [KS-test] *p* = 0.03, Table S1) in LCa-EGFP–expressing cells (Figure 2B) and showed no decrease in σ2-EGFP–expressing cells (Figure 2D). Thus, we conclude that elevated TfnR concentrations result in more efficient CCP maturation, but that TfnR concentration is not rate-limiting for CCV formation. The approximately 2-fold increase in efficiency of CCP maturation in the presence of an approximately 40-fold increase in cargo concentration points to the existence of other limiting factors and is consistent with the saturation of endocytic efficiency observed biochemically (Figure S3A).

### Cargo-Enhanced CCP Maturation Requires AP2 Complexes

To further probe the role of AP2 adaptors in pit maturation, we decreased cellular AP2 levels by approximately 50% through short-term small interfering RNA (siRNA)-mediated knockdown of the μ2-subunit (unpublished data) [21]. AP2 depletion reduced the densities of all classes of pits (from 0.477 ± 0.012 μm^2^ in control to 0.276 ± 0.069 μm^2^ in the knockdown), although the relative contributions of the three populations and their lifetimes remained unchanged (Figure 2A and 2B). This suggests that AP2-dependent nucleation events lead proportionally to both short-lived abortive and productive pits, implying a precursor/product relationship. In contrast to control cells, TfnR overexpression in AP2-depleted cells did not lead to an increase in the relative contribution of the productive population (Figure 2A). We conclude that the increase in CCP maturation efficiency with increased cargo concentration requires AP2 and/or is limited by AP2 concentration.

### Dynamin Controls Rate-Limiting Early Stages of CME

Given that TfnR/cargo concentration only marginally affects lifetimes of CCPs, we next investigated which factor(s) might regulate this aspect of CCP dynamics. The self-assembling GTPase dynamin has been suggested to play a dual role in CME, both as a regulator and/or fidelity monitor during early, rate-limiting steps in endocytosis, and as a well-documented component of the fission apparatus late in CCV formation [22–24]. Dynamin is recruited along with clathrin and AP2 [2], and the early activities of dynamin presumably occur while it is associated with coated pits in its unassembled state, utilizing its basal GTP binding and hydrolysis activities [22,23]. In contrast, dynamin function in membrane fission requires its self-assembly and assembly-stimulated GTPase activities [22,23,25] and occurs subsequent to a burst of recruitment at late stages of CCP formation [5–7]. We sought direct evidence for dynamin's dual role in CME by examining the effects of well-characterized dynamin mutants on CCP dynamics by siRNA-mediated knockdown of dynamin-2 and reintroduction of siRNA-resistant wild-type (WT) or mutant dynamin-1. Because dominant-negative dynamin mutants block endocytosis and lead to clustering of nonproductive CCPs (unpublished data), we focused our analysis on three well-characterized hypoactive dynamin mutants: (1) Dyn1K694A is impaired in self-assembly and hence specifically in assembly-stimulated GTPase activity [22]. Overexpression of dyn1K694A was shown to increase rates of CME [22]. (2) Dyn1S61D exhibits WT GTP binding, but reduced basal and assembly-stimulated GTP hydrolysis rates [26]. Overexpression of dyn1S61D was shown to reduce rates of CME [26]. (3) Dyn1T141A exhibits reduced GTP binding in the unassembled, basal state, but increased basal GTP hydrolysis rates. It also exhibits a slight increase in assembly-stimulated GTPase activity and overexpression of dyn1T141A slightly increases the rate of CME [26].

**Figure 4:**
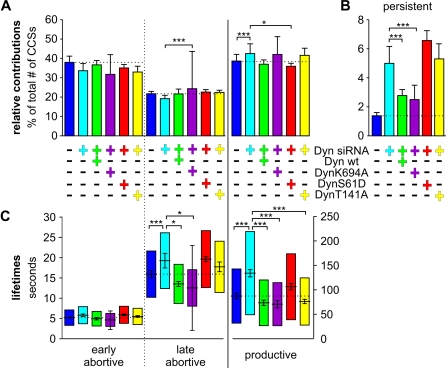
Dynamin Regulates CCP Maturation and CCV Budding Cells were either left untreated (ctrl) or transfected with dynamin-2–specific siRNA, after which we reintroduced siRNA-resistant WT or mutant dynamin-1 as indicated. (A) The relative contributions of CCP subpopulations are largely unaffected by the treatments shown. (B) The offset fraction of very long-lived CCPs, termed “persistent,” is increased in siRNA-treated cells. (C) The lifetimes of CCP subpopulations are significantly affected by siRNA treatment and overexpression of dynamin WT and dynamin mutants. A single asterisk (*) and triple asterisks (***) indicate confidence levels of *p* < 0.05 and *p* < 10^−4^, respectively (KS-test). The number of CCP trajectories (*n*) and cells (*k*) for each condition are: Dyn-2 siRNA only (*n* = 13,410, *k* = 18); siRNA+dyn1WT (*n* = 37,280, *k* = 24); siRNA+dyn1K694A (*n* = 8,263, *k* = 7); siRNA+dyn1S61D (*n* = 28,436, *k* = 25); siRNA+dyn1T141A (*n* = 22,550, *k* = 18).

The effects of dynamin-2 knockdown and expression of these mutants on the relative contributions of the different subpopulations were minor compared to the effect of cargo overexpression (Figure 4A); however, after dynamin-2 knockdown we now detect a substantial increase in the fraction of long-lived (>10 min), “persistent” CCPs that were negligible in control BSC1 cells (Figures 4B, S1E, and S1F). Reintroduction of WT dynamin-1 (dyn1WT) or dyn1K694A reduced the number of persistent CCPs, whereas reintroduction of dyn1S61D or dyn1T141A mutants increased their numbers.

In contrast to cargo overexpression, perturbations of dynamin function had dramatic effects on the lifetimes of CCP subpopulations. Knockdown of dynamin-2 to approximately 17% of endogenous levels (see Figure S3E and S3F) significantly (KS-test, *p* < 10^−10^) increased the characteristic lifetime of productive CCPs (Figure 4C and Table S1), confirming a role for dynamin as a rate-limiting factor in CCV formation. Dynamin-2 knockdown also increased the characteristic lifetime of late abortive CCPs (Figure 4C). This observation is consistent with its proposed role during early stages in CCV formation.

After knockdown of dynamin-2, overexpression of dyn1WT significantly decreased the characteristic lifetime of the productive population (KS-test, *p* < 10^−7^, Figure 4C, Table S1), again consistent with dynamin controlling rate-limiting steps in CME. In addition, overexpression of dynWT decreased the lifetime of late abortive coated pits, suggesting a role for dynamin as a fidelity monitor that initiates rejection and disassembly of nonviable CCPs. Overexpression of the self-assembly–impaired dyn1K694A mutant also significantly decreased the characteristic lifetime of both the productive and late abortive subpopulations (KS-test, *p* < 10^−16^, *p* < 10^−2^, respectively; Figure 4C). These data suggest that unassembled dynamin functions early, that this assembly-impaired mutant stimulates CME by enhancing the rate of CCP maturation, and that dynamin self-assembly and subsequent assembly-stimulated GTPase activities per se are not rate-limiting for CCV formation [22]. Overexpression of the GTPase-defective dyn1S61D was unable to fully restore the rate of productive CCV formation in dynamin-2 siRNA-treated cells (Figure 4C), and indeed increased the lifetimes of late abortive and productive CCPs even when overexpressed in the presence of endogenous dynamin-2 (unpublished data). Lastly, overexpression of dyn1T141A was unique in that it differentially affected the abortive and productive lifetimes (Figure 4C): the lifetimes of abortive CCPs remain slightly increased relative to control cells (KS-test, *p* = 0.08), whereas the lifetime of productive CCPs became shorter (KS-test, *p* = 0.008). Together with the dyn1S61D findings, these results demonstrate that GTP binding and hydrolysis in the basal state are required for an early surveillance function of dynamin and that the basal rate of GTP hydrolysis might be rate-limiting for maturation towards the productive CCP subpopulation. These data provide direct evidence that dynamin plays a dual role in CCP maturation and vesicle budding.

### Dynamin Regulates CCP Maturation

**Figure 5:**
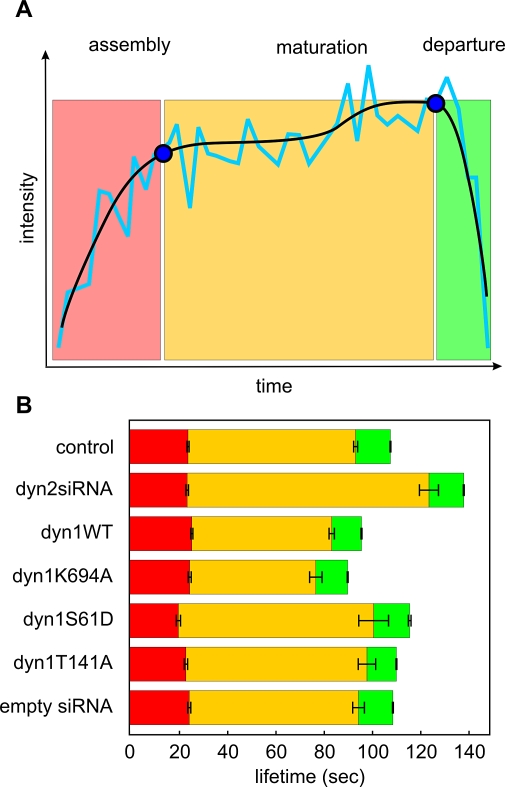
Distinct Phases in CCP Maturation Defined by Intensity Time Courses (A) Example of typical intensity time course of an isolated productive CCP, fitted with a smoothing spline. The time course is divided into three phases—termed assembly, maturation, and departure—based on the points where the slope of the spline drops under a given threshold (20% of the maximum). (B) Bar graph of corresponding phase lengths (averaged over all extracted trajectories) for different treatments. Error bars indicate standard error of the mean. The number of CCP trajectories (*n*) for each condition are: control (*n* = 905); Dyn-2 siRNA only (*n* = 249); siRNA+dyn1WT (*n* = 907); siRNA+dyn1K694A (*n* = 194); siRNA+dyn1S61D (*n* = 140); siRNA+dyn1T141A (*n* = 248); empty siRNA (*n* = 345).

To provide further evidence for dynamin's role in CCP maturation, we extracted the intensity time courses of the LCa-EGFP signal during CCP maturation. For this purpose, we focused our analysis on a subset of long-lived, isolated CCPs, which are highly likely (>99%) to represent productive events. The typical intensity time course is skewed (see example in Figure 5A) and can be divided into three distinct phases: (1) an “assembly phase” corresponding to the initial fast increase of signal intensity, which occurred during the first approximately 20 s of detectable clathrin lattice assembly; followed by (2) a “maturation phase,” during which LCa-EGFP signal intensity plateaus or increases only moderately; followed by (3) a “departure phase,” characterized by a sudden final drop of signal intensity. To measure the duration of these phases, the intensity time courses of individual CCP trajectories were fitted with a smoothing spline (Figure 5A) to identify the points where the approximated slope drops below a set threshold. siRNA-mediated knockdown of dynamin-2 markedly increased the duration of the maturation phase (*t*-test, *p* < 10^−10^), without significantly altering assembly or departure phases (see Table S3). These data are consistent with a role for dynamin in regulating rate-limiting steps in CCP maturation and with the fact that dynamin-mediated membrane fission is not rate-limiting for CME [22]. The average length of the assembly and departure phases (Figure 5B) were largely unaffected by reintroduction of the various dynamin mutants. In contrast, the length of the maturation phase increased and decreased depending on the GTP binding and hydrolysis activities of the reintroduced dynamin. Specifically, re-expression of dyn1WT or dyn1K694A mutants decreased, whereas re-expression of GTPase-defective dyn1S61D or dyn1T141A increased the duration of the maturation phase. These findings support a role for the basal GTP binding and hydrolysis activities of dynamin in CCP maturation.

## Discussion

We report a comprehensive and unbiased analysis of CCP dynamics in living cells. This was accomplished using a new particle tracking algorithm that defines the correspondence between CCP images in consecutive frames based on spatial and temporal global optimization [10], which allowed us to reliably follow the fate of CCPs in areas of both low and high pit density. The algorithm incorporates a gap closing scheme that permitted tracking of faint and temporarily unstable CCPs. The performance of this algorithm was extensively validated for its application to CME analysis [10]. To capture the sub-second scale events of CCP formation and the much slower events of CCP maturation, we merged lifetime data from high frequency, shorter time-lapse videos with lower frequency, longer time-lapse videos. Thus, we tracked tens of thousands of both short-lived and long-lived species for each experimental condition without biasing the selection of CCPs to a subpopulation with a specific characteristic, e.g., only isolated or bright pits. The large sample number enables application of statistical model-selection methods to determine the minimum number of subpopulations necessary to accurately fit the measured lifetime distribution. Indeed, application of these statistical methods requires *n* > 5,000, a criterion met in each of our analyses, but which greatly exceeds the 100–600 selected events previously assessed in other studies [1–5]. Importantly, subsequent molecular perturbations identified certain conditions in which the contribution of subpopulations to the overall lifetime distribution changed while their time constants were unaffected, whereas other conditions left contributions unchanged while significant shifts occurred in the time constants. This indicates the orthogonality of the two parameters we extracted and establishes that they can be independently determined to probe distinct mechanistic aspects of CCP maturation.

In control BSC1 cells expressing LCa-EGFP, we detected three CCP subpopulations (early abortive, late abortive, and productive), with distinct time constants (∼5 s, ∼15 s, and ∼90 s, respectively). A previous analysis of CCP dynamics in the same cells suggested an average lifetime of approximately 46 s, assuming a single population of CCPs [2]. Taking into consideration the different contributions of these subpopulations to the entire ensemble of CCPs, we obtain a value of 39 s, consistent with this previous data. We suspect that the slightly lower ensemble lifetime in our measurements may be associated with a more systematic exclusion of the very faint, short-lived early abortive CCPs in the previous study [2]. The longer CCP lifetimes (60–90 s) consistently reported by others [1,6] reflect selection and analysis of a single subset of typically productive CCPs.

The functional assignment of productive CCPs rested on the agreement of the lifetimes of the long-lived subpopulation with biochemical rates of TfnR uptake, as well as with the lifetimes of LCa-EGFP structures tracked manually in quasi-simultaneous TIRF- and epifluorescent images showing CCV internalization. Therefore, we interpret the short-lived CCPs to represent abortive events, but it is also conceivable that they could represent clathrin-coated intracellular structures, e.g., endosomes, that transiently move as visitors through the evanescent field of the TIRF microscope. However, for the following reasons, we think this possibility is unlikely: (1) Although we occasionally observe fast-moving visitors in LCa-EGFP–labeled cells, their displacement between consecutive frames is so much above average that particle correspondences are generally outside the tracking algorithm's self-adaptive search range [10]. Thus, the trajectories of visitors are typically fragmented into short sub-trajectories (less than three frames), similar to trajectories associated with false-positive detections. To exclude both types of false structures from the lifetime analysis, trajectories shorter than five frames were removed from the dataset (see Materials and Methods). (2) Early and late abortive events detected with LCa-EGFP are also detected by our statistical model selection following σ2-EGFP, a selective marker of plasma-membrane–associated CCPs. In addition, the early abortive σ2-labeled CCPs have virtually the same lifetime as early abortive LCa-labeled CCPs, giving us confidence that these are bona fide plasma membrane–associated structures. (3) The relative contributions of both abortive and productive CCPs are affected by transferrin receptor overexpression, and their lifetimes are affected by dynamin. There is no reason why these parameters should be affected for visitors unrelated to CCPs. (4) The strongest indication that the vast majority of the structures we have studied are plasma membrane–associated CCPs comes from the AP2 depletion experiments in which the numbers of all three subpopulations are proportionally decreased. This would not be expected if the shorter-lived species were derived from internal membranes. Furthermore, AP2 depletion prevents the shift to productive CCPs induced by TfnR overexpression. Thus, although it is possible that there is a minor contamination of CCPs by clathrin-coated internal membrane vesicles or clathrin-coated nonendocytic structures, their contribution appears not to be significant enough to affect our findings.

Having identified three kinetically distinct subpopulations of CCPs, we next showed that their relative distributions and lifetimes could be affected by systematically manipulating cargo concentration, adaptor protein levels and the level and activity of the GTPase dynamin. Based on the shifts in contribution and lifetime of the three subpopulations we propose that CME might be governed by an endocytosis checkpoint or restriction point, which is regulated, in part, by dynamin. The following observations support the existence of this checkpoint: (1) the identification of abortive and productive CCPs (see also [2]), (2) the finding that AP2-containing (and presumably cargo-enriched) CCPs are more likely to be productive, (3) the finding that cargo load enhances the efficiency of CCV formation leading to more productive CCPs at the expense of abortive ones, and (4) the finding that this effect of cargo concentration is dependent on or limited by AP2 adaptor concentrations.

Progression through a restriction or checkpoint requires the tight interaction of a monitor and an activator system. As the major GTPase involved in CME, dynamin was a prime candidate to regulate the endocytosis checkpoint. Two models have been proposed for dynamin function in endocytosis: as a regulatory molecule [27] and as a component of the fission machinery [28–30]. However, these are not mutually exclusive, and recent data support aspects of both [23–26]. Our data on the effects of dynamin depletion and dynamin mutants on CCP dynamics provide several lines of evidence that unassembled Dyn•GTP acts early and controls progression through the endocytosis checkpoint: (1) siRNA reduction of dynamin decreases the rate of both productive CCV formation and turnover of abortive CCPs, whereas overexpression of WT dynamin accelerates each of these rates; (2) a mutant defective in self-assembly (K694A) that is predicted to increase cellular levels of unassembled Dyn•GTP further increases the rates of abortive CCP turnover; and (3) dynamin GTPase domain mutants predicted to be defective in basal GTP binding (T141A) or hydrolysis (S61D) selectively reduce the rates of abortive CCP turnover. Importantly, these conclusions rely on the use of well-characterized, hypomorphic dynamin mutants that mildly alter the kinetics of CME, yet have robust and readily detectable effects when assessed by large-scale image analysis. Strong dominant-negative dynamin mutants that stop CME lead to the accumulation of aggregated CCPs, thus limiting their usefulness for mechanistic interpretation.

Dynamin is also positioned to act as a monitor of factors that satisfy restriction point requirements through its many SH3 domain–containing binding partners. These have additional domains that recognize coat proteins (e.g., amphiphysin, SNX9,) membrane curvature (e.g., amphiphysin, endophilin, SNX9), and/or cargo molecules (e.g., SNX9, grb2, TTP). It is known that these proteins can differentially affect dynamin's basal GTPase activity and assembly properties [31–33]. Hence, they provide a potential mechanism for regulating dynamin function in response to these parameters of CCP maturation. Dynamin has also been shown to interact with auxilin and hsc70 [34], thus providing a potential mechanism for the dynamin-dependent turnover of abortive CCPs that we have observed.

**Figure 6:**
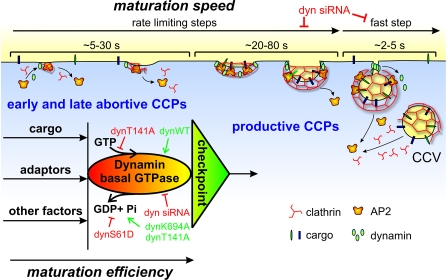
Model of Endocytosis Checkpoint That Controls Clathrin-Coated Pit Maturation CCVs are formed from productive CCPs, which have undergone a maturation process. An endocytosis checkpoint gates the maturation process, and CCPs that do not progress beyond this restriction point abort by sequential disassembly of AP2 and clathrin. Progression through the restriction point is dependent on the concentration of cargo receptors, AP2 adaptors, and probably other factors. The GTP binding and hydrolysis activities of unassembled dynamin, as revealed by analysis of dynamin GTPase mutants, control the rate and extent of progression through this checkpoint. Dynamin assembly and assembly-stimulated GTPase activities required at late stages of CCV formation are not rate-limiting. See text for details.

A model describing these results is illustrated in Figure 6. In this model, productive CCP formation is a stochastic event initiated by the cargo-independent association of AP2 at the plasma membrane, which nucleates clathrin assembly. If a critical mass of the additional factors required for CCP stabilization is not reached during this assembly phase, these structures, which correspond to early and late abortive CCPs, fail to pass through the restriction point and disassemble. We propose that dynamin regulates the checkpoint and controls entry into and progression through the CCP maturation phase. The basal GTP binding and hydrolysis activities may enable unassembled dynamin to function either as a sensor or timer of CCP assembly, through its SH3 domain–containing partners, and thus directly or indirectly control the termination or progression of early endocytic intermediates. Further work will be needed to test this hypothesis and to determine the mechanistic details of how dynamin may monitor CCP assembly and maturation.

In sum, we propose that the presence of sufficient cargo, a threshold concentration of AP2 adaptors and perhaps other parameters such as the recruitment of endocytic accessory factors, acquisition of membrane curvature, etc., are detected by dynamin to signal progression beyond the endocytosis checkpoint. Whereas three kinetically distinct subpopulations are detected with statistical significance in our analyses, the lifetime distributions—particularly of the productive population—remain very broad. Thus, we expect that there are other aspects of functional heterogeneity and other factors regulating the endocytosis checkpoint masked within this subpopulation. Future studies involving mild perturbation of other endocytic accessory factors together with comprehensive quantitative analysis of CCP dynamics should provide further mechanistic insight into this functional heterogeneity.

## Materials and Methods

### Total internal reflection fluorescence microscopy.

TIR-FM was performed on BSC1 monkey kidney epithelial cells stably expressing rat brain clathrin LCa-EGFP or the AP2 rat brain σ2-adaptin fused to EGFP (provided by Dr. T. Kirchhausen, Harvard Medical School, and described in [2]) using a 100 × 1.45 NA objective (Nikon) mounted on a Nikon TE2000U inverted microscope (Nikon). CCP lifetimes range from a few seconds to several minutes. To fully capture this range of dynamics would require image sampling over minutes at a high frame rate (<1 s). Such exposure leads to significant photobleaching and also substantially impairs cell viability, both affecting the accuracy of lifetime measurements. To avoid these problems, we applied a multi-timescale imaging approach. For each experimental condition, three to nine videos with a frame rate of 0.4 s/frame were acquired over >200 s, and five to 21 videos with a frame rate 2 s/frame, each from different cells, were acquired over approximately 10 min, using a 14-bit mode operated Hamamatsu Orca II-ERG camera.

### Image and lifetime analysis.

CCPs were detected using à-trous wavelet transform decomposition of the image [35]. Tracking of CCP was accomplished using spatially and temporally global particle assignment described in detail elsewhere [10]. The histograms of CCP lifetimes extracted from the two TIR-FM video categories were merged for the final lifetime analysis by normalizing the relative contribution of the CCP population with a lifetime in the range 30 to 50 s. Thus, the integrals of the measured lifetime probability density functions *g_i,_*_[0.4]_ from all *N*_[0.4]_ videos sampled at 0.4 s/frame and the integrals of the measured lifetime functions *f_j,_*_[2]_ from all *M*_[2]_ videos sampled at 2 s/frame were set to equal values:


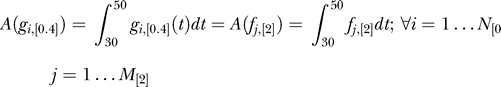


From the merged lifetime histograms, the underlying distributions of multiple CCP populations with different lifetime dynamics were extracted using both parametric and nonparametric model selection as described in detail in Text S1.

For the intensity analysis, we extracted the trajectories of CCPs that were long-lived (>60 s) and isolated (no nearest neighbors within six pixels); this criterion ensured that the chosen CCPs were in fact “productive,” i.e., that they underwent full maturation and internalization, and that there was no crossover to neighbors, both in terms of the physical material of the CCP and in terms of the tracked CCP trajectories, both of which can lead to artifacts in the intensity time course.

### Online supplementary material.

Text S1 contains a more detailed description of methods including particle tracking, lifetime analysis, cell culture, adenoviral infection, cell fractionation, western blotting, immunofluorescence, siRNA transfection, and biochemical measurement of endocytic uptake. Tables S1 and S2 summarize the mean lifetimes and relative contributions of CCP subpopulations in each experimental condition. Table S3 summarizes the intensity phase data.

Figure S1 shows data relating to the validation of tracking and lifetime analysis. Figure S2 shows three statistical methods used to identify CCP subpopulations. Figure S3 shows the effect of TfnR overexpression on (1) endocytosis efficiency and (2) subcellular distribution of AP2 and clathrin, and a western blot of dynamin knock-down and corresponding quantification. Videos S1–S6 show examples of particle detection and tracking in simulated videos (Video S6) and those obtained imaging live cells (Videos S1–S5).

## Supporting Information

Figure S1 Validation of Tracking and Lifetime Analysis (A) Videos with CCP density and signal-to-noise similar to real data were simulated with a Dirac lifetime distribution (ground truth; lifetime = 90 frames). Detection and tracking of the CCPs reproduces a Dirac peak at 90 frames, but also generates artificially short lifetimes due to detection or gap-closing failures.
(B) Videos were simulated with a Gaussian lifetime distribution (τ_0_ = 90, σ = 5). Notice that the ground truth distribution jitters because of the limitations of the random number generator. A fit to the lifetime distribution of the detected and tracked trajectories yields τ_0_ = 89.5, σ = 5.3, statistically indistinguishable from the ground truth (KS-test *p* = 0.36)
(C) Videos were simulated with a lifetime distribution reflecting an exponential decay process (τ = 40). A fit to the lifetime distribution of the detected and tracked trajectories yields τ = 39.8, after correction for the cutoff probability of long trajectories due to the finite video length. Ground truth and reconstructed distribution are statistically indistinguishable (KS-test *p* = 0.99).
(D) Histogram showing the frequency in measured lifetime data of closed gaps of varying lengths.
(E) Original lifetime histogram (top) and cumulative lifetime histogram (bottom) of an example dataset.
(F) Zoom-in of long lifetimes showing the cutoff of the cumulative histogram (dark blue, solid line) at 300 s and the continuation of the fitted cumulative probability density function (light blue, dashed line). Offset b300 is determined by the relative occurrence of objects of lifetime >300 s. The fit of lifetime distribution models explicitly accounts for a plateau offset bin, which corresponds to the offset of the fitted function at infinity. The plateau offset defines the “persistent” population of long-lived pits that are functionally distinct from the productive population.
(234 KB TIF)

Figure S2 Separation of Subpopulations with Different Lifetime Distributions (A) BIC test on fit of cumulative lifetime histogram with a mixture-model of Weibull distributions. Blue: fit of lifetime (independent variable) versus cumulative frequency (dependent variable); green: fit of rank (independent variable) versus lifetime (dependent variable).
(B) KS-test on residuals of mixture-model fit with different number of subpopulations. Red horizontal line defines *p*-value of a 95% confidence interval for significantly different residual distributions as determined by bootstrap analysis. All three statistical methods identify three CCP subpopulations in control cells.
(C) BIC test on different mixture models with specified shape constants for different numbers of trajectories contributing to the lifetime histogram.
(157 KB TIF)

Figure S3 Characteristics of Clathrin-Mediated Endocytosis in BSC1 Cells and Effects of Overexpression of Transferrin Receptors (A) Receptor-mediated endocytosis of transferrin is saturable. BSC1 were infected with adenovirus coding for human TfnR in a tet-repressible system. After overnight induction in the absence of tet, the internalization of transferrin was followed in cells overexpressing the transferrin receptor (cargo o/x) and compared to control cells, cultured in the presence of tet (ctrl). After incubation for the indicated times at 37 °C, the amount of internalized transferrin was determined and expressed as a percentage of total surface-bound TfnR.
(B) Manual lifetime quantification of productive CCPs. Lifetimes of 450 readily detectable CCPs were determined from the first appearance to their departure. Endocytosis was confirmed by detecting sequential departure from the TIRF and then epifluorescent field.
(C and D) Membrane recruitment of coat components. Cells have been cultured as in (A) and analyzed by western blot after subcellular fractionation, S indicates the cytosolic fraction; P the membrane fraction. β-tubulin served as loading control. The cell fractionation experiment has been repeated at least three times, and a typical experiment is shown.
(D) Bar graph of quantitation showing the percentage of membrane-associated clathrin and AP2 in control and TfnR-overexpressing cells. Error bars represent standard deviations of three independent experiments.
(E and F) si-RNA–mediated Dynamin knock-down. Immunoblot showing dynamin-2 content in BSC1 cells transfected with control siRNA (ctrl) or dynamin-2–specific siRNA (+ dyn siRNA) and quantification of a representative western blot. Actin served as loading control.
(647 KB TIF)

Table S1Mean Lifetimes of Clathrin-Coated Pits Detected by TIR-FMNote: *k* and *τ* are the shape factor and the time constant (characteristic lifetime) of the population's corresponding Weibull function *f*(*t*) = *a* ·(*k*/*τ*)*^k−^* ^1^ · exp(−*t*/τ)*^k^*; ± *t*50 denotes the interval on both sides of τ containing 50% of the data in the distribution, *i.e.* is the jackknifed error (standard deviation [sd]) of the time constant τ, representing cell-to-cell variation. τ*_m_* is the mean lifetime (expectancy value) of the distribution, where τ*_m_* = τ · Γ(1 + 1/*k*), so that τ*_m_* = τ for *k* = 1 and 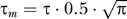 for*k* = 2. *n_traj_* is the number of trajectories measured for the condition, *n_cells_* the number of cells.
(115 KB DOC)

Table S2 Relative Contributions of Clathrin-Coated Pit Subpopulations Detected by TIR-FM (78 KB DOC)

Table S3 Intensity Phases of Productive CCPs Detected by TIR-FM (55 KB DOC)

Text S1 Details of Methodology Methodology includes cell culture, microscopy, biochemistry, particle detection and tracking, validation, lifetime analysis, and statistical methods. (156 KB DOC)

Video S1 Clathrin (LCa-EGFP), Frame Rate 0.4 s Left panel: original image with overlaid detected particle positions and one-frame dragtail of trajectory (red). Right panel: binary image (mask) of à-trous particle detection results with overlaid particle positions (red circles) and closed gaps in trajectory (blue crosses). Note that contrast is adjusted automatically, which may reduce visibility of dimmer objects. Image size 10 × 10 μm, pixel size approximately 67 nm.
(6.37 MB MOV)

Video S2 Clathrin (LCa-EGFP), Frame Rate 2 s Left and right panels are as in Video S1.
(6.35 MB MOV)

Video S3 AP2 (σ2-EGFP), Frame Rate 2 s Left and right panels are as in Video S1.
(4.98 MB MOV)

Video S4 Clathrin (LCa-EGFP) Zoom-In, Frame Rate 2 s Left and right panels are as in Video S1.
(2.33 MB MOV)

Video S5 AP2 (σ2-EGFP) Zoom-In, Frame Rate 2 s Left and right panels are as in Video S1.
(2.09 MB MOV)

Video S6 Simulation of CCS Video with Intensity Parameters Chosen to Resemble Measured Data Lifetimes correspond to superposition of three subpopulations as for clathrin control conditions. Left and right panels are as in Video S1.
(2.19 MB MOV)

## Abbreviations

BIC: Bayesian Information Criterion
CCP: clathrin-coated pit
CCV: clathrin-coated vesicle
CME: clathrin-mediated endocytosis
EGFP: enhanced green fluorescent protein
KS-test: Kolmogorov-Smirnov test
LCa: light chain a
siRNA: small interfering RNA
tet: tetracycline
TfnR: transferrin receptor
TIR-FM: total internal reflection fluorescence microscopy
WT: wild type
