# Supplementary material for: Cargo and Dynamin Regulate Clathrin-Coated Pit Maturation
Source: PLoS Biol. 2009 Mar 17;7(3):e1000057. doi: 10.1371/journal.pbio.1000057 (PMC2656549; doi:10.1371/journal.pbio.1000057)
Supplement: Supplementary file 1 [file pbio.1000057.sd001.doc]

**Supplementary Material: *D. Loerke, M. Mettlen et al., Cargo and dynamin regulate clathrin-coated pit maturation***

Online Methods

**Cell culture**

BSC1 monkey kidney epithelial wild type (wt) cells or BSC1 cells stably expressing rat brain clathrin light chain-EGFP or the AP2 rat brain σ2-adaptin fused to EGFP [1] (LCa-EGFP and σ2-EGFP, respectively) were generously provided by Dr. T. Kirchhausen, Harvard Medical School, Boston. NIH 3T3 cells (mouse fibroblasts) stably expressing LCa-DsRed were kindly provided by W. Almers, Vollum Institute, Oregon Health and Sciences University. BSC1 wt and HeLa cells were transfected with 2 μg LCa-Tomato (kindly provided by Dr. T. Kirchhausen) using Lipofectamine 2000 (Invitrogen) transfection reagent for 4 h in Optimem (Gibco) according to the manufacturer’s instructions. All cells were grown in DMEM supplemented by 20 mM HEPES, 10 µg/ml streptomycin, 66 µg/ml penicillin 10% (v/v) fetal calf serum (FCS, HyClone) and 0.5 mg/ml geneticin (Invitrogen) under 5% CO_2_ at 37°C.

Adenoviral infection, total internal reflection fluorescence microscopy (TIR-FM) and immunofluorescence

For live cell imaging, BSC1 cells were plated at a density of 9 x 10³ cells/cm² on 22x22 mm glass coverslips (refractive index: 1.523; Corning) and co-infected with adenoviruses coding for the protein of interest (i.e. TfnR, WT or mutant dynamins) and adenoviruses coding for a tetracycline repressible transcription activator. After overnight incubation in the presence (1000 ng tet/ml; control cells) or absence of tetracycline (to allow protein overexpression), through-the-objective TIR-FM was performed as described previously [2]. Briefly, cells were preincubated for >30 min with imaging medium (DMEM, devoid of phenol red and containing 10 mM HEPES and 5% fetal bovine serum, pH 7.5) supplemented or not with tetracycline at 37°C. Coverslips were then mounted on a slide with two strips of double-sided tape spaced 1 cm apart. The volume between the strips of tape and the coverslip was filled with corresponding imaging medium and sealed with a 1:1:1 mixture of Vaseline, lanolin, and paraffin. For imaging, the slides were rapidly transferred to a prewarmed microscope stage (37°C; controlled with a custom-modified stage incubator; 20–20 Technologies). Four or five cells were imaged per coverslip. The theoretical depth of the evanescent field (*d*) was <100 nm. However, given that the decay of the evanescent field is exponential, and not absolute, brighter objects deeper than *d* can be observed by TIR-FM [3]. Exposure times were 80–200ms, depending on the frame rate and the intensity of the signal.

For immunofluorescence, cells were cultured as described above. To visualize TfnR overexpression, cells were pre-incubated for 60 min at 37°C in DMEM without FCS for depleting serum transferrin, and then 10 µg/ml of Alexa-Fluor-568 conjugated transferrin (Invitrogen) was loaded at 15°C for 25 min. Incubation at this temperature prevents internalization of transferrin, while the transferrin concentrates on coated pits. Transferrin-loaded cells were then observed by Epi-fluorescence microscopy, using identical acquisition settings.

siRNA treatment

For siRNA treatment, RNA oligos (Dharmacon) were used that target the following sequences: µ2: AAGUGGAUGCCUUUCGGGUCA [4]; dynamin-2: CCGAATCAATCGCATCTTC and GACATGATCCTGCAGTTCA [5]. BSC1 cells expressing LCa-EGFP were plated on coverslips or 60 mm Petri dishes at a density of either 8.3 x 10³ (for single-round µ2-siRNA treatment) or 2.6 x 10³ cells/cm² (for double-round dynamin-2-siRNA treatment). After overnight incubation, cells were washed with Opti-MEM (Invitrogen) and transfected once (µ2) or twice (at 24-h intervals; dynamin-2) with siRNA oligos (40 nM final concentration) using HiPerFect transfection reagent as delivery agent (Qiagen), according to manufacturer's recommendations. Cells were placed in ordinary culture medium 8 h after transfection and processed for TIR-FM or Western blot analysis one/two days after initial transfection. Rescue of dynamin-2 knock-down cells was performed by adenoviral-mediated expression of HA-dynamin-1, as described above.

**Cell fractionation and Western blotting**

For Western blots to detect dynamin, clathrin, AP2 or TfnR expression levels, cells grown in 60 mm dishes were washed twice with PBS and harvested in PBS containing 5 mM EDTA. Low speed cell pellets were then either processed for cell fractionation (see below), or directly lysed in 200 µl lysis buffer (50 mM Tris-base, 1 % (v/v) Nonidet P-40, 150 mM NaCl, 2 mM EGTA, 2 mM Na_3_VO_4_, 0.25% (w/v) deoxycholic acid, 10 mM NaF, and protease inhibitor cocktail [Roche Diagnostics], pH 7.4).

For cell fractionation, cell pellets were gently resuspended in 250 µl fractionation buffer (25 mM sucrose, 1 mM MgCl_2_, 2 mM EGTA, 25 mM Hepes, pH 7.4) and lysed by 3 cycles of freezing/thawing in liquid N_2_. Whole cell lysates were centrifuged at 110,000 g in a TLA100.2 Beckman rotor (Beckman Coulter Inc.) for 30 min. The soluble fraction (cytosolic fraction "S") was collected and pellets (membrane fraction "P") were resuspended in 250 µl fractionation buffer. Equal amounts of "S" and "P" fractions were solubilized in 0.1 % (w/v; final concentration) Triton X-100 and sonicated.

For Western blots, samples were separated using SDS–polyacrylamide gel electrophoresis, transferred to a nitrocellulose membrane (Whatman), blocked for 1 h at room temperature in blocking buffer (20 mM Tris-base, 15 mM NaCl, 5 mM EDTA, 0.5 % (v/v) Tween-20, 5% (w/v) milk, pH 7.4), incubated overnight with primary antibodies: anti-human TfnR (clone H68.4; Zymed), anti-dynamin-2 (C-18, Santa Cruz), anti-β-tubulin (clone Tub2.1; Sigma-Aldrich), anti-clathrin (TD.1; hybridoma) and anti-α-adaptin (clone 100/2; Sigma-Aldrich), and revealed by HRP-labeled secondary antibodies and enhanced chemiluminescence. For a rigorous quantitation, chemiluminescent blots were analyzed by an Alpha Innotech FluorChem SP image station (Alpha Innotech). Values were obtained by AlphaEase FC image analysis software (version 4.1.0; Alpha Innotech).

**Estimation of surface bound/endocytic uptake of transferrin receptors**

Cells were washed 3 x in PBS, harvested at room temperature in PBS containing 5 mM EDTA, pelleted in the presence of MgCI_2_ (1 mM final concentration), and resuspended in PBS^++++^ (1 mM CaCI_2_, 1 mM MgCI_2_, 0.2% BSA (w/v), and 5 mM glucose). Cells were incubated in the presence of biotinylated human transferrin (BSST; 8 µg/ml final concentration) on ice for 45 min to allow binding, followed by 3 gentle washes (resuspension/sedimentation) to remove non-bound BSST. Aliquots of cells were either kept on ice and lysed as described below (to determine the amount of surface exposed TfnR) or warmed to 37°C for the indicated times to measure endocytic uptake. Endocytosis was stopped by returning cells to ice and non-internalized BSST was masked by successive treatment with avidin and biocytin (Sigma). Finally, cells were lysed in blocking buffer (2% BSA (w/v), 1% Triton-X100 (v/v), 0.1% SDS (w/v), 1 mM EDTA, 50 mM NaCl and 10 mM Tris-HCl, pH 7.4).

To measure surface bound and intracellular BSST (BSST binds to TfnRs in a 1:1 stoichiometry), known amounts of BSST (standard curve) and cell lysates were loaded into 96-well ELISA plates (NUNC) coated with anti-human Tf antibodies and incubated overnight at 4°C. After extensive washes with dPBS and blocking buffer, plates were incubated with streptavidin−horseradish peroxidase conjugate (1:5,000; Roche) for 1 h. HRP activity was measured by a stopped colorimetric assay using ortho-dianisidine as a substrate. Light absorption at 490 nm was determined with a Bio-Stack microplate reader (Bio-Tek) and all values were corrected by light absorbance at 650 nm and normalized by the total cell protein content (bicinchoninic acid assay).

Particle detection

Particle detection was performed with a home-written software package that built, in essence, on the spot extraction methods based on multiscale products of significance-thresholded wavelet coefficients originally proposed by Olivo-Marin [6]. This method combines two strengths that are critical for reliable and unbiased detection of CCPs. First, the decision as to whether an image feature is significant, i.e. whether it constitutes with high probability a CCP, is made locally. Thus, particle detection is invariant with respect to global intensity variations due to unevenness of the TIR-FM illumination, unspecific and diffuse concentration changes in LCa-EGFP and σ2-EGFP, and uneven distributions of CCPs. Second, the selection of significant CCPs is size-invariant. Thus, the algorithm finds at the same time diffraction-limited spots (the majority of CCP images) as well as temporary fusions of CCP images.

In brief, these two properties are achieved by wavelet decomposition of the raw image. As proposed in [6], we used the B-spline approximation of *a-trous* wavelets. Wavelet decomposition results in a series of wavelet planes $W_{i}(x,y), i=1...J$ that emphasize image structures of increasingly larger size ($W_{J}$ the largest). Local maxima in wavelet planes tend to propagate across size scales if they are associated with a significant image feature, while they do not if they are caused by noise. Thus, by per pixel-multiplication of all wavelet planes $P(x,y)=\prod_{i=1}^{J} W_{i}(x,y)$, true image features associated with CCPs are enhanced while suppressing noise. To further stabilize the feature selection in this so-called correlation image, Olivo-Marin suggested separately applying a threshold that extracts pixels significantly above the statistical variation of the background of the specific plane to each wavelet plane. Per pixel-multiplication is applied on the thresholded wavelet planes resulting in a modified correlation image $P_{t}(x,y)$. Subsequently, pixels are selected as members of spots if $P_{t}(x,y)>\lambda$, where λ is a user-defined detection level. We found that the result is sensitive to the setting of this level and requires visual inspection for each movie to achieve a satisfactory result. To allow efficient, automatic processing of the large number of movies analyzed in this study and to minimize user subjectivity we implemented a parallel thread of image thresholding: First, we used the individually thresholded wavelet planes to reconstruct a noise-suppressed image $S(x,y)$. Then, we calculated for each pixel the local average and variance in a 9x9 neighborhood ($E_{[9x9]}[S]$ and $Var_{[9x9]}[S]$, respectively), as well as the global average ($E[S]$). A pixel in $S(x,y)$ is considered a member of a CCP image if

$$\left(P_{t}(x,y)>\lambda\right) and \left(S(x,y)>E[S]\right) and \left(S(x,y)>E_{[9x9]}[S]+0.5\cdot Var_{[9x9]}[S]\right)$$

With these additional conditions λ could be set to a non-restrictive level, i.e. no need for fine-adjustment from movie to movie, while variations in the image quality between movies were accounted for by global and local conditions imposed on the noise-suppressed image. In particular, the combination of the first two criteria was effective in eliminating isolated pixels. CCP images were then reconstructed by connected-component labeling of all the pixels passing the above set of criteria. Each CCP was considered a particle for subsequent particle tracking.

Particle tracking

Particle tracking was performed with a home-written software package tuned to analyze dense particle fields with heterogeneous dynamics; this tracking system employs global combinatorial optimization to solve *both* the problem of particle association between consecutive frames and the problem of linking partial trajectories, i.e. gap-closing. Details on the algorithm and can be found in Jaqaman et al. [7]. The performance of the tracking algorithm for our system is validated in Supplementary Figure S1. For a visual impression of the robustness of the method we refer to Supplementary Movies S1-S5, which show examples of the tracking results for both the LCa-EGFP and σ2-EGFP fluorescence tags, and in both slow and fast acquisition movies. In its implementation for CCP tracking, the algorithm relies on two control parameters: a CCP’s maximal velocity, and the optimal maximum time window for gap closing. This was determined via detailed analysis of the stability of the lifetime distributions[7] and set to 8 frames; this corresponds to a maximum permissible gap length of 7 frames. In practice, however, most closed gaps (>70%) were ≤3 frames (Supplementary Figure S1D, see also validation of tracking and lifetime analysis below).

**Lifetime analysis**

The trajectories extracted by the tracking software fall into two different categories: trajectories that appear and disappear during the movie; and trajectories that are cut off by the beginning or end of the movie, i.e. objects that are present in the first and/or last frame of the movie. For the former, the lifetime is equal to the length of time between appearance and disappearance. For the latter, the precise lifetime is unknown. Given a movie of length $m$, and assuming that the object will appear with equal probability at any possible time point, the probability that a newly appeared object of lifetime *t* will be cut off by the end of the movie is $c(t,m)=(1+t)/m$, where $c(t>m)=1$. Conversely, the probability of observing this object’s trajectory in its entirety is $e(t,m)=1-c(t,m)$. Thus, if the actual number of trajectories of length $t_{i}$ (*i* frames) is $R(t_{i})$ (where $R(t_{i})=n\cdot\int_{t_{i}-0.5\cdot\Delta t}^{t_{i}+0.5\cdot\Delta t} pdf$ with $n$ the number of observations, $\Delta t$ the frame rate, and *pdf* the probability density function of the CCP lifetime distribution) then the number of trajectories of length $t_{i}$ that can be observed in their entirety is $O(t_{i})=R(t_{i})\cdot e(t_{i})$. Thus, for lifetimes that are an appreciable fraction of the movie length, and for lifetime distributions with a wide spread, the observed lifetime distribution $O(t_{i})$ has to be corrected with $cf=1/e(t_{i})=m/(m-1-t_{i})$ to yield the actual distribution $R(t_{i})=O(t_{i})\cdot cf$. Normalization of this distribution then yields the measured cumulative lifetime histogram $\hat{r}(t_{i})=R(t_{i})/\sum_{i} R(t_{i})$. This cumulative lifetime histogram corresponds to the lifetime distribution function – the complement of the survival function – in reliability theory. To fit any model $f(t)$ of the CCP lifetime distribution to the measured data we minimize the objective function $\sum_{i=1}^{T} \left(\hat{r}(t_{i})-\int_{t=0}^{T} f(t)\right)^{2}$ i.e. the unknown model parameters in $f(t)$ are determined such that the cumulative histogram of particle lifetimes and the cumulative probability density function are in best match between 0 and the cut-off time T (Supplementary Fig. S1E). For our analyses we chose T = 300s, i.e. half of the typical maximum movie length. For longer lifetimes $T>m/2$, more than half of the trajectories are cut off ($c(T,m)>0.5$), so that the proportion of ‘speculative’ trajectories would exceed 50%. For the purpose of normalization we determine the function offset at 300s $b_{300}$ from the number of *all* objects of lifetime >300s (Supplementary Fig. S1F), regardless of whether their entire trajectory is observed or not. Importantly, this also includes stationary pits, i.e. CCPs that are visible over the entire course of the movie. In addition to the relative contributions and lifetimes of the 3 functional subpopulations, the fit includes a plateau parameter $b_{\inf}$, which corresponds to the function offset at infinity. This plateau of ‘persistent’ pits is negligible under control and cargo o/x conditions, where it contributes ~1%. This indicates that under these conditions, most of the very long-lived or even stationary pits (which produce the $b_{300}$ offset) in fact constitute the tail end of the broad lifetime distribution of productive CCPs. In contrast, the fraction of persistent pits increases significantly in treatments that inhibit dynamin function.

**Validation**

To evaluate the accuracy of our lifetime analysis, we simulated random-walk trajectories of objects with assumed lifetime distributions (“ground truth”). Based on these trajectories, we generated noisy image stacks that resembled our experimental images in all parameters (Supplementary Movie S6); pixel size = 67nm; object size (FWHM of Gaussian) = 250nm; background intensity = 510±5cts; average intensity of objects = 56cts above background; object-to-object intensity variation = 8cts, frame-to-frame intensity variation of an object = 10%. Simulated movies were then subjected to the complete detection/tracking/lifetime analysis, and the resulting estimated lifetimes were compared to the ground truth of the simulation. For a distribution of objects with identical lifetime of 90 frames (Supplementary Fig. S1A), the majority of lifetimes are recovered correctly, while some trajectories are cut short by detection or gap-closing failures. In the histogram, these failures create a small artifact for very short lifetimes (≤3 frames). Importantly, while the population of very short lifetimes makes a significant contribution to the overall lifetime histogram, the fraction of these particles per frame is <3 %. This value corresponds to the rate fraction of false positives during the statistical selection of particles. While temporary losses of CCPs due to false negatives are corrected by gap closing, false positives typically generate trajectories with very short lifetimes. Accordingly, in our analyses we discarded trajectories shorter than 4 frames. To a first approximation, the rate of false positive detection is independent of the frame rate. Hence, in our analysis the cutoffs for fast and slow sampling were equal.

For Gaussian ground truth, both the mean and the spread of the lifetimes are reproduced with high accuracy (p=0.36 in KS-test comparing the estimated to the ground truth lifetime distribution, see Supplementary Fig. S1B). In an exponential distribution with a spread exceeding the length of the simulated movie, the measured lifetime histogram decays more quickly than the ‘ground truth’ distribution (Supplementary Fig. S1C), which is primarily caused by the higher cutoff probability of long trajectories. Accordingly, the input and output distributions are significantly different (KS-test p<10^-4^). To compensate for this effect, lifetimes were corrected for cutoff as described above, after which the exponential lifetime distribution is reproduced with high accuracy (KS-test p=0.99).

It should be noted that spatially and temporally global tracking intrinsically favors short over long gaps. Hence, although the maximum allowed gap length is 7 frames, the majority of closed gaps is <3 frames (see Supplementary Fig. S1D). The length distribution of closed gaps is primarily determined by detection noise. Thus, the distributions are virtually identical for movies with fast and slow sampling. Occasionally, a true CCP disappearance event can be erroneously linked to a true CCP appearance event; this occurs more frequently in movies with slow sampling, because the longer gap window in terms of absolute time (as opposed to frames) makes the re-appearance of a new CCP within the vicinity of a disappearance event more likely. The gap length distribution shows that the frequency of long gaps (> 5 frames, i.e. > 10 s in slow movies) is increased by merely 3% in slow movies (Supplementary Fig. S1D). Accounting for the contribution of long gaps to the total number of gaps, the fraction of erroneous long gap closings is << 1% of the total number of gaps.

**Statistical Identification of Subpopulations**

To identify the contributions of distinct CCP subpopulations with different dynamics to the measured lifetime distribution, we fitted the cumulative lifetime histograms with a superposition of multiple distributions. Each individual distribution *j* was described by a Weibull function:

$$f_{i}(t)=a_{i}\cdot(k/\tau_{i})^{k-1}\cdot\exp(-t/\tau_{i})^{k}$$

where $a_{i}$ is the amplitude, $\tau_{i}$ the time constant, and $k_{i}$ the shape parameter [8]. For *k=2*, the Weibull defines a Rayleigh distribution (R), describing a maturation process, for *k=1*, it defines an exponential distribution (E), describing a particle decay process; values of *k* in the interval *1<k<2* describe mixed behavior.

The number of significant subpopulations contributing to a measured lifetime distribution is *a priori* unknown. To estimate the number of distributions and their parameters, we used multiple approaches:

1.) A popular tool for model selection is the minimization of the value of the Bayesian information criterion (BIC). The BIC value weighs the goodness of the fit against the number of model parameters: $BIC=(n\cdot\log(RSS/n))+p\cdot\log(n)$, where *n* denotes the sample size (number of points being fitted), *RSS* the sum of the squared residuals of the fit, and *p* the number of free parameters [9]. Accordingly, minimization of the BIC implies simultaneous minimization of the residuals (first term) and the number of parameters necessary to achieve a satisfactory model fit (second term). In other words, the model with the minimal BIC uses the largest statistically justifiable number of model parameters to optimally describe the measured data without overfitting. However, this approach requires some assumptions about the error distribution of the measured data. While the BIC can be formulated generally for any a priori known error distribution [9], the simplest and easiest to implement formulation above intrinsically assumes that the fitting residuals are normally distributed. This assumption is inherently invalid when fitting the original lifetime histogram where binning effects rather yield binomially distributed errors. Thus, we performed mixture model fitting on the cumulative histogram, where the error more closely resembles a normal distribution (data not shown). In this approximation, the BIC minimization yields 3 significant sub-populations (Supplementary Fig. S2A).

2.) To transform BIC-based model selection into a domain where the error more closely approximates a normal distribution, the lifetime distribution function above (lifetime as the independent variable, cumulative frequency as the dependent, error-perturbed variable) was inverted to define a curve with the histogram rank as the independent variable and the original lifetime measurement as the dependent, error-perturbed variable. While the noise of lifetime measurements is also not normally distributed *per se*, the fitting of inverted cumulative histograms minimizes the residuals in the domain of the actual measurement. Particularly for faint image signals, where the magnitude of shot noise is comparable or less than camera noise, the assumption of a normally distributed lifetime error is a reasonable approximation. Interestingly, BIC-based model selection in the transformed cumulative lifetime histograms yields the same 3 significant populations (Supplementary Fig. S2A).

3.) To circumvent the need for assumptions about the type of residual distribution we also used a model selection scheme based in a non-parametric test. In this approach, we tested the residuals of the fit with *i* populations against the residuals of the fit with *j* populations using a Kolmogorov-Smirnov-test (KS-test). The significance level of this test was determined via bootstrap analysis of the residuals of a fit with 3 sub-populations, choosing the 95% confidence of the KS-test between random samples from the same pool of residuals. The results show that residuals are significantly altered when increasing the number of sub-populations from 1 or 2 to a model with 3 sub-populations (Supplementary Fig. S2B). However, any additional sub-population beyond 3 does not significantly change the residual distribution. Thus, both BIC-based model selection relying on the approximation of normally distributed fitting residuals, and comparison of the unknown residual distributions as sub-populations are added by non-parametric statistical tests yield the same outcome. This suggests that the inaccuracy in assuming normally distributed residuals has no substantial effect on the model selection.

While these results demonstrate that the lifetime data are best described by a mixture model of 3 Weibull populations, they do not prove that a Weibull function is the optimal description of each subpopulation *per se*. However, a comparison of the goodness-of-fit of three-population mixture models with different distribution types (3 independent distributions with 3 parameters $a_{i},k_{i},\lambda_{i}$ each) fitted to the cumulative lifetime histogram confirmed the Weibull distribution as by far the best approximation of the tested models. The goodness-of-fit is expressed as the sum of the squared residuals. Conversion of this value into the standard deviation of an individual residual from the Weibull-based mixture model indicates that the model approximates the normalized frequency values of the cumulative histogram by >99%

| Type of distribution | Probability density function | Goodness-of-fit  (Sum of squared residuals) |
| --- | --- | --- |
| Weibull | $\left(\frac{k}{\lambda}\right)\cdot\left(\frac{t}{\lambda}\right)^{k-1}\exp(-(t/\lambda)^{k})$ | 0.0027 |
| Chi square  (which includes Maxwell) | $\frac{2^{1-k/2}t^{k-1}\exp(-t^{2}/2)}{\Gamma(k/2)}$ | 0.3467 |
| Lognormal | $\frac{1}{tk\sqrt{2\pi}}\exp\left[-\frac{(\ln(t)-\lambda)^{2}}{2k^{2}}\right]$ | 0.2964 |
| Gamma distribution | $t^{k-1}\frac{\exp(-t/\lambda)}{\Gamma(k)\lambda^{k}}$ | 0.6570 |

We found that the sensitivity of the mixture-model fit to the shape parameter *k* is low, i.e. a large range of values for *k* generates equally well-fitting models. As expected for a low sensitivity, parameter *k* correlated strongly with the time constants of the mixture models, precluding direct comparison of the latter for different conditions. Thus, we constrained the shape parameters to the best-performing combination of integer values. The best-performing integer values agreeing for clathrin and AP2 movies were $k_{i}=[2,2,1]$, i.e. a combination of two Rayleigh and one exponential distribution.

The discrimination power of the model selection depends on the accurate sampling of the entire spread of possible lifetimes. Thus, the analysis described in this paper critically relied on a pool of trajectories large enough to distinguish subpopulations that may even have similar time constants (like the ‘early abortive’ and the ‘late abortive’ CCPs). To estimate the number of necessary data points we tested by simulation the stability of the model selection. We simulated lifetimes of three subpopulations using time constants, relative contributions and image noise in agreement with experimental data, and then tested how the BIC value of a given model changed with an increasing number of trajectories added to the analysis (Supplementary Fig. S2C). We found that the analysis of samples containing n < 2,000 trajectories was insufficient to retrieve the correct models. Only for samples containing n > 5,000 trajectories did the correct mixture of three distributions outcompete alternative models.

Online References

1.	Ehrlich M, Boll W, Van Oijen A, Hariharan R, Chandran K, Nibert ML, Kirchhausen T (2004) Endocytosis by random initiation and stabilization of clathrin-coated pits. Cell 118: 591-605.

2.	Yarar D, Waterman-Storer CM, Schmid SL (2005) A dynamic actin cytoskeleton functions at multiple stages of clathrin-mediated endocytosis. Mol Biol Cell 16: 964-975.

3.	Rappoport JZ, Simon SM, Benmerah A (2004) Understanding living clathrin-coated pits. Traffic 5: 327-337.

4.	Motley A, Bright NA, Seaman MN, Robinson MS (2003) Clathrin-mediated endocytosis in AP-2-depleted cells. J Cell Biol 162: 909-918.

5.	Huang F, Khvorova A, Marshall W, Sorkin A (2004) Analysis of clathrin-mediated endocytosis of epidermal growth factor receptor by RNA interference. J Biol Chem 279: 16657-16661.

6.	Olivo-Marin J-C (2002) Extraction of spots in biological images using multiscale products. Pattern Recognition 35: 1989-1996.

7.	Jaqaman K, Loerke D, Mettlen M, Kuwata H, Grinstein S, Schmid SL, Danuser G (2008) Robust single-particle tracking in live-cell time-lapse sequences. Nat Methods 5: 695-702.

8.	Weibull W (1951) A statistical distribution function of wide applicability. J Appl Mech 18: 293-297.

9.	Burkard KE, Cela E (1999) Linear assignment problems and extensions. In: Du DZ, Pardalos PM, editors. Handbook of combinatorial optimization. Kluwer Academic Publishers. Dordrecht, The Netherlands. pp. 75-149.
