# Supplementary material for: Cargo and Dynamin Regulate Clathrin-Coated Pit Maturation
Source: PLoS Biol. 2009 Mar 17;7(3):e1000057. doi: 10.1371/journal.pbio.1000057 (PMC2656549; doi:10.1371/journal.pbio.1000057)
Supplement: Supplementary file 2 [file pbio.1000057.st001.doc]

**Supplementary Table 1: Mean lifetimes of CCPs**

|  |  | $\tau$ | $\pm t\text{50}$ | $j\text{.}e\text{.}$ | $n_{\text{traj}}$ | $n_{\text{cells}}$ |
| --- | --- | --- | --- | --- | --- | --- |
| **LCa-EGFP**  **BSC1 cells** |  |  |  |  |  |  |
| control | early abortive | 5.2 | 1.9 | 0.1 | 43,658 | 23 |
|  | late abortive | 15.9 | 5.7 | 1 |  |  |
|  | productive | 86.9 | 55.3 | 5.8 |  |  |
| TfnR o/x | early abortive | 6.7 | 2.4 | 0.8 | 23,626 | 18 |
|  | late abortive | - | - | - |  |  |
|  | productive | 80.0 | 50.9 | 9.8 |  |  |
| control siRNA | early abortive | 5.6 | 2 | 0.2 | 9,376 | 10 |
|  | late abortive | 16.2 | 5.8 | 1.3 |  |  |
|  | productive | 87.5 | 55.6 | 12.2 |  |  |
| μ2 siRNA | early abortive | 5.6 | 2 | 0.2 | 29,106 | 27 |
|  | late abortive | 16.8 | 6 | 1 |  |  |
|  | productive | 92.4 | 58.8 | 8.2 |  |  |
| μ2 siRNA + TfnR o/x | early abortive | 5.3 | 1.9 | 0.4 | 11,071 | 12 |
|  | late abortive | 15.9 | 5.7 | 1.9 |  |  |
|  | productive | 78.6 | 50 | 20.7 |  |  |
| dyn siRNA | early abortive | 5.7 | 2.1 | 0.4 | 13,410 | 18 |
|  | late abortive | 19.2 | 6.9 | 1.8 |  |  |
|  | productive | 130.9 | 83.2 | 12.1 |  |  |
| dyn siRNA + dynwt o/x | early abortive | 5.0 | 1.8 | 0.2 | 37,280 | 24 |
|  | late abortive | 13.5 | 4.8 | 0.6 |  |  |
|  | productive | 73.2 | 46.5 | 4.8 |  |  |
| dyn siRNA + dynK694A o/x | early abortive | 4.6 | 2.3 | 1.7 | 8,236 | 7 |
|  | late abortive | 12.5 | 10.6 | 4.5 |  |  |
|  | productive | 69.9 | 9.7 | 44.4 |  |  |
| dyn siRNA + dynS61D o/x | early abortive | 5.9 | 2.1 | 0.2 | 28,436 | 25 |
|  | late abortive | 19.6 | 7 | 0.6 |  |  |
|  | productive | 105.9 | 67.3 | 6.6 |  |  |
| dyn siRNA + dynT141A o/x | early abortive | 5.5 | 2 | 0.2 | 22,550 | 18 |
|  | late abortive | 17.7 | 6.3 | 1.2 |  |  |
|  | productive | 75.8 | 48.2 | 4.5 |  |  |
| **LCa-tomato**  **BSC1 cells** |  |  |  |  |  |  |
| control | early abortive | 5.3 | 1.9 | 0.2 | 25,453 | 20 |
|  | late abortive | 15.3 | 5.5 | 0.9 |  |  |
|  | productive | 101.3 | 64.4 | 7 |  |  |
| **σ2-EGFP**  **BSC1 cells** |  |  |  |  |  |  |
| control | early abortive | 4.8 | 1.8 | 0.4 | 22,252 | 21 |
|  | late abortive | 8.4 | 3 | 1.8 |  |  |
|  | productive | 71.5 | 45.5 | 6 |  |  |
| TfnR o/x | early abortive | 5.2 | 1.9 | 0.8 | 14,100 | 16 |
|  | late abortive | - | - | - |  |  |
|  | productive | 71.5 | 45.5 | 7.4 |  |  |

**Supplementary Table 1: Mean lifetimes of CCPs** (continued)

| **LCa-tomato Hela cells** |  |  |  |  |  |  |
| --- | --- | --- | --- | --- | --- | --- |
| control | early abortive | 6.1 | 2.2 | 0.1 | 11,152 | 17 |
|  | late abortive | 20.9 | 7.5 | 0.4 |  |  |
|  | productive | 93.3 | 59.3 | 8.1 |  |  |

| **LCa-dsRed NIH3T3 cells** |  |  |  |  |  |  |
| --- | --- | --- | --- | --- | --- | --- |
| control | early abortive | 6.1 | 2.2 | 0.1 | 21,356 | 19 |
|  | late abortive | 20.6 | 7.4 | 0.6 |  |  |
|  | productive | 114.9 | 73.1 | 4.8 |  |  |
