# Supplementary material for: Cargo and Dynamin Regulate Clathrin-Coated Pit Maturation
Source: PLoS Biol. 2009 Mar 17;7(3):e1000057. doi: 10.1371/journal.pbio.1000057 (PMC2656549; doi:10.1371/journal.pbio.1000057)
Supplement: Supplementary file 3 [file pbio.1000057.st002.doc]

**Supplementary Table 2: Relative contribution of CCPs**

|  |  | relative contribution | jackknife  error |
| --- | --- | --- | --- |
| **LCa-EGFP**  **BSC1 cells** |  | (% of total) |  |
| control | early abortive | 38.1 | 3.1 |
|  | late abortive | 21.9 | 1.4 |
|  | productive | 38.6 | 3.4 |
| TfnR o/x | early abortive | 28.8 | 7.6 |
|  | late abortive | - | - |
|  | productive | 67.9 | 7.9 |
| control siRNA | early abortive | 37.0 | 2.1 |
|  | late abortive | 20.7 | 2.4 |
|  | productive | 41 | 2.8 |
| μ2 siRNA | early abortive | 33.6 | 1.4 |
|  | late abortive | 19.5 | 1.3 |
|  | productive | 44.4 | 2.2 |
| μ2 siRNA + TfnR ox | early abortive | 31.5 | 4.2 |
|  | late abortive | 22.8 | 8.9 |
|  | productive | 43.9 | 5.7 |
| dyn siRNA | early abortive | 33.5 | 3.8 |
|  | late abortive | 19.2 | 1.7 |
|  | productive | 42.4 | 5 |
| dyn siRNA + dynwt o/x | early abortive | 36.7 | 2.4 |
|  | late abortive | 21.5 | 2.4 |
|  | productive | 39.0 | 3.5 |
| dyn siRNA + dynK694A o/x | early abortive | 31.6 | 10.3 |
|  | late abortive | 24 | 19.7 |
|  | productive | 41.9 | 9.4 |
| dyn siRNA + dynS61D o/x | early abortive | 35 | 1.6 |
|  | late abortive | 22.6 | 0.8 |
|  | productive | 35.8 | 1.6 |
| dyn siRNA + dynT141A o/x | early abortive | 32.9 | 3.2 |
|  | late abortive | 20.2 | 1.2 |
|  | productive | 41.6 | 3.7 |
| **LCa-tomato**  **BSC1 cells** |  |  |  |
| control | early abortive | 28.4 | 2.0 |
|  | late abortive | 18.6 | 1.0 |
|  | productive | 51.7 | 2.5 |
| **σ2-EGFP**  **BSC1 cells** |  |  |  |
| control | early abortive | 20.8 | 10.1 |
|  | late abortive | 18.8 | 4.2 |
|  | productive | 59.3 | 10.1 |
| TfnR o/x | early abortive | 22.6 | 6.7 |
|  | late abortive | - | - |
|  | productive | 76.8 | 5.6 |

**Supplementary Table 2: Relative contribution of CCPs (continued)**

| **LCa-tomato Hela cells** |  |  |  |
| --- | --- | --- | --- |
| control | early abortive | 33.3 | 2 |
|  | late abortive | 20.6 | 1.2 |
|  | productive | 40.2 | 1.9 |

| **LCa-dsRed**  **NIH3T3 cells** |  |  |  |
| --- | --- | --- | --- |
| control | early abortive | 29.4 | 0.8 |
|  | late abortive | 20.9 | 0.7 |
|  | productive | 46.3 | 1.7 |
