# Supplementary material for: Cargo and Dynamin Regulate Clathrin-Coated Pit Maturation
Source: PLoS Biol. 2009 Mar 17;7(3):e1000057. doi: 10.1371/journal.pbio.1000057 (PMC2656549; doi:10.1371/journal.pbio.1000057)
Supplement: Supplementary file 4 [file pbio.1000057.st003.doc]

**Supplementary Table 3: Intensity phases**

| **Clathrin Intensities** | control | dyn2 siRNA | dyn2 siRNA + WT | dyn2 siRNA + K694A | dyn2 siRNA + S61D | dyn2 siRNA + T141A | empty siRNA |
| --- | --- | --- | --- | --- | --- | --- | --- |
| **n** | 905 | 249 | 907 | 194 | 140 | 248 | 345 |
| **Phase 1: Assembly** | | | | | | | |
| mean | 26.52 | 25.9 | 28.18 | 26.56 | 21.55 | 25.12 | 27.81 |
| Standard deviation | 12.58 | 11.38 | 13.27 | 12.34 | 10.42 | 11.73 | 12.71 |
| Standard error of the mean | 0.42 | 0.72 | 0.44 | 0.89 | 0.7 | 0.74 | 0.77 |
| p-value of T-test against control | - | 0.6207 | 0.01331 | 0.5017 | <10e-4 | 0.08410 | 0.4331 |
| p-value of T-test against empty | - | 0.2915 | 0.2895 | 0.9679 | <10e-4 | 0.3242 | - |
| **Phase 2: Maturation** | | | | | | | |
| mean | 66.12 | 97.22 | 54.59 | 49.53 | 90.83 | 72.06 | 55.8 |
| Standard deviation | 43.4 | 58.45 | 45.05 | 38.23 | 66.56 | 54.74 | 42.33 |
| Standard error of the mean | 1.44 | 3.7 | 1.5 | 2.74 | 4.46 | 3.48 | 2.57 |
| p-value of T-test against control | - | <10e-10 | <10e-6 | <10e-6 | 0.0089 | 0.0033 | 0.8822 |
| p-value of T-test against empty | - | <10e-10 | <10e-4 | <10e-4 | 0.0441 | 0.0238 | - |
| **Phase 3: Departure** | | | | | | | |
| mean | 15.34 | 15.32 | 13.13 | 14.18 | 15.93 | 13.16 | 14.52 |
| Standard deviation | 6.78 | 5.8 | 5.42 | 5.16 | 7.36 | 6.26 | 5.31 |
| Standard error of the mean | 0.23 | 0.37 | 0.18 | 0.37 | 0.49 | 0.4 | 0.32 |
| p-value of T-test against control | - | 0.9651 | <10e-10 | 0.02320 | 0.2478 | 0.07253 | 0.9028 |
| p-value of T-test against empty | - | 0.8898 | <10e-6 | 0.0407 | 0.2606 | 0.1172 | - |
